# The microRNA-10a/ID3/RUNX2 axis modulates the development of Ossification of Posterior Longitudinal Ligament

**DOI:** 10.1038/s41598-018-27514-x

**Published:** 2018-06-15

**Authors:** Chen Xu, Hao Zhang, Wei Gu, Huiqiao Wu, Yuanyuan Chen, Wenchao Zhou, Baifeng Sun, Xiaolong Shen, Zicheng Zhang, Yue Wang, Yang Liu, Wen Yuan

**Affiliations:** 1 Spine Center, Department of Orthopaedics, Changzheng Hospital, Second Military Medical University, 415th Feng Yang Road, Shanghai, 200003 P.R. China; 2 Department of Orthopedic Surgery, Sixth People’s Hospital Affiliated to Shanghai Jiao Tong University, 800th Yi Shan Road, Shanghai, 200233 P.R. China; 3 Research Center of Developmental Biology, Second Military Medical University, 800th Xiang Yin Road, Shanghai, 200433 P.R. China; 4 Administration Office for Graduate Students, Changhai Hospital, Second Military Medical University, 168th Chang Hai Road, Shanghai, 200433 P.R. China

## Abstract

Ossification of the posterior longitudinal ligament (OPLL) presents as pathological heterotopic ossification of the spinal ligaments. However, its underlying molecular mechanism is still unclear. Our previous findings suggested that altered microRNA regulatory network are critical for the development of OPLL. Here, we set out to unveiling the detailed mechanism of those altered OPLL-specific microRNAs. We screened a set of differentially expressed OPLL-specific microRNAs from the previous sequencing data and showed that microRNA-10a actively modulates the ossification of posterior ligament cells *in vitro*. Using a tissue-engineered scaffold grown from 4-week-old BALB/c homozygous nude mice, we found that altered microRNA-10a expression in posterior ligament cells indeed affected the heterotopic bone formation *in vivo*. Furthermore, computational analysis showed that the negative ossification regulator ID3 is a functional target gene of microRNA-10a, and its expression was also significantly altered during microRNA-10a modulation both *in vitro* and *in vivo*. Also, we have demonstrated that the ossification promoting function of microRNA-10a requires ID3, as ID3 actively inhibits RUNX2. Thus, we identified a critical role for highly altered OPLL-specific microRNA-10a in regulating the development of OPLL by modulating the ID3/RUNX2 axis.

## Introduction

Ossification of the posterior longitudinal ligament (OPLL) is a common spinal disease that often causes severe injuries and compressions to the spinal cord and nerve roots. However, the exact etiology and mechanisms are far from clear. Pathologically, OPLL is characterized by ectopic ossification in the posterior ligament of the spine, the growing bony spurs can cause myelopathy, radiculopathy or even quadriplegia through direct injury or compression to the spinal cord or nerve roots^1^.

Although previous studies have suggested that the causes of OPLL are largely related to abnormal bone and mineral metabolism, the underlying mechanism and regulatory networks have not been fully elucidated^2–5^. miRNAs are small (17–22 nucleotides), single stranded, non-coding RNAs that are important regulators of gene expression in many biological processes and diseases^6^. Recent findings showed that various miRNAs can regulate ossification. For example, miR-690 activated by RUNX2 positively regulates RUNX2-induced osteogenic differentiation by inactivating the NF-κB pathway via the downregulation of subunit p65^7^, whereas TNF-α-induced NF-kB activation upregulates microRNA-150-3p and inhibits osteogenesis of mesenchymal stem cells by targeting β-catenin^8^. These results suggest that osteogenesis is tightly controlled by miRNAs, and miRNA regulation may be an upstream mediator of the osteogenic process. However, such regulatory networks are not elucidated in OPLL.

A recent study by our group defined OPLL-specific microRNAs (miRNAs) using high-throughput miRNA sequencing and revealed that their predicted regulatory network was closely related to ossification development^9^. However, their specific roles in OPLL development needs further identification. In our preliminary research, we found that differentially expressed OPLL-specific miRNAs may have effects on osteogenic differentiation of posterior ligament cells. In this work, we set out to investigate the function and underlying mechanism of these highly altered OPLL-specific miRNAs using both *in vitro* and *in vivo* technics. Through computational analysis, we try to elucidate the function of four most altered OPLL-specific miRNAs, and found that miR-10a-3p showed most significant effect. Using computational prediction and validation, we found that miR-10a-3p can actively suppress the translation of ID3, a RUNX2 negative regulator, to promote osteogenic differentiation and mineral deposition of posterior ligament both *in vitro* and *in vivo*. Our work here demonstrated the important role of miR-10a-3p in the ossification process of OPLL, and revealed its mechanism through the ID2/RUNX2 axis, which may further contributes to the understanding of OPLL development.

## Results

### Highly altered microRNAs are potential regulators of ossification

**Figure 1:**
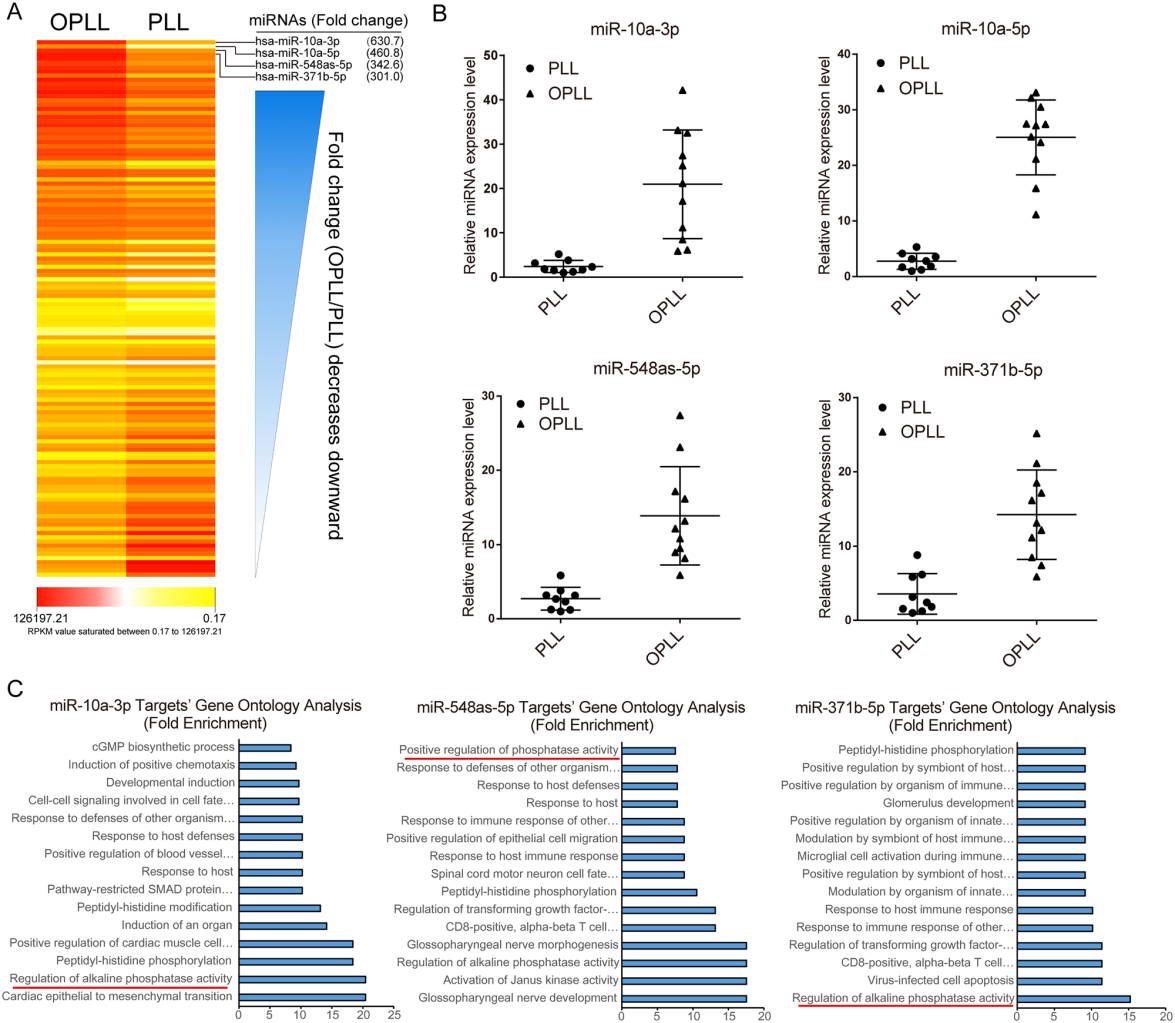
Highly Altered microRNAs are potential ossification regulators. (**A**) The sequenced miRNA data of GSE69787 in Gene Expression Omnibus database were reanalyzed in a fold change order (OPLL/PLL). Average Reads Per Kilobases per Millionreads (RPKM) for each group were mapped shown in a heatmap style. The magnitude of deviation from the median is represented by color saturation. (**B**) The expression level of top 4 differentially expressed miRNAs were validated in ligament tissues between OPLL (n = 11) and PLL (n = 9) using real-time PCR. All values in every miRNA analysis were compared to the least one in each test and each dot represents the average expression of triplicates in one subject. The error bars represents ±S.D. **P < 0.01, t-test. (**C**) Gene Ontology analysis of the predicted targets of miR-10a-3p, miR-548as-5p and miR-371b-5p. The initial prediction results were further aligned with the transcription data of GSE69787 to find correlated targets.

Previously, we reported the expression changes of microRNAs (miRNAs) along with their predicted regulatory network in OPLL compared with PLL (None ossified normal longitudinal ligament)^9^. Here, we further elucidated the involvement of these miRNAs in the development of OPLL. We hypothesized that more differentially expressed miRNAs may have active roles in OPLL development. Thus, we organized the differentially expressed miRNAs by fold change and selected the top four for further analysis (Fig. 1A, Supplementary Data 1). Next, we validated the expression changes of these miRNAs using real-time polymerase chain reaction (PCR) analysis of OPLL (n = 11; twelve samples were used, but one failed to extract high quality RNAs) and PLL (n = 9) tissue samples and showed that the results were consistent with the next generation sequencing (NGS) data (Fig. 1B). To assess their role in regulating posterior ligament ossification, we used previously established miRNA targeting networks and examined the functions of these predicted targets using Gene Ontology (GO) analysis. Of the four miRNAs examined (miR-10a-5p yielded 0 predicted targets in the previously established regulation network), miR-10a-3p, miR-548as-5p and miR-371b-5p’s predicted targets all showed significant enrichment in the GO terms “regulation of alkaline phosphatase activity” and “skeletal system development”, while “positive regulation of ossification” was enriched only in miR-10a-3p’s targets (P < 0.05, fold enrichment = 5.1, Fig. 1C and Supplementary Data 2). These results indicate a role of miR-10a-3p, miR-548as-5p and miR-371b-5p in regulating the ossification process of OPLL.

### MicroRNA-10a actively modulates ossification of ligament cells

**Figure 2:**
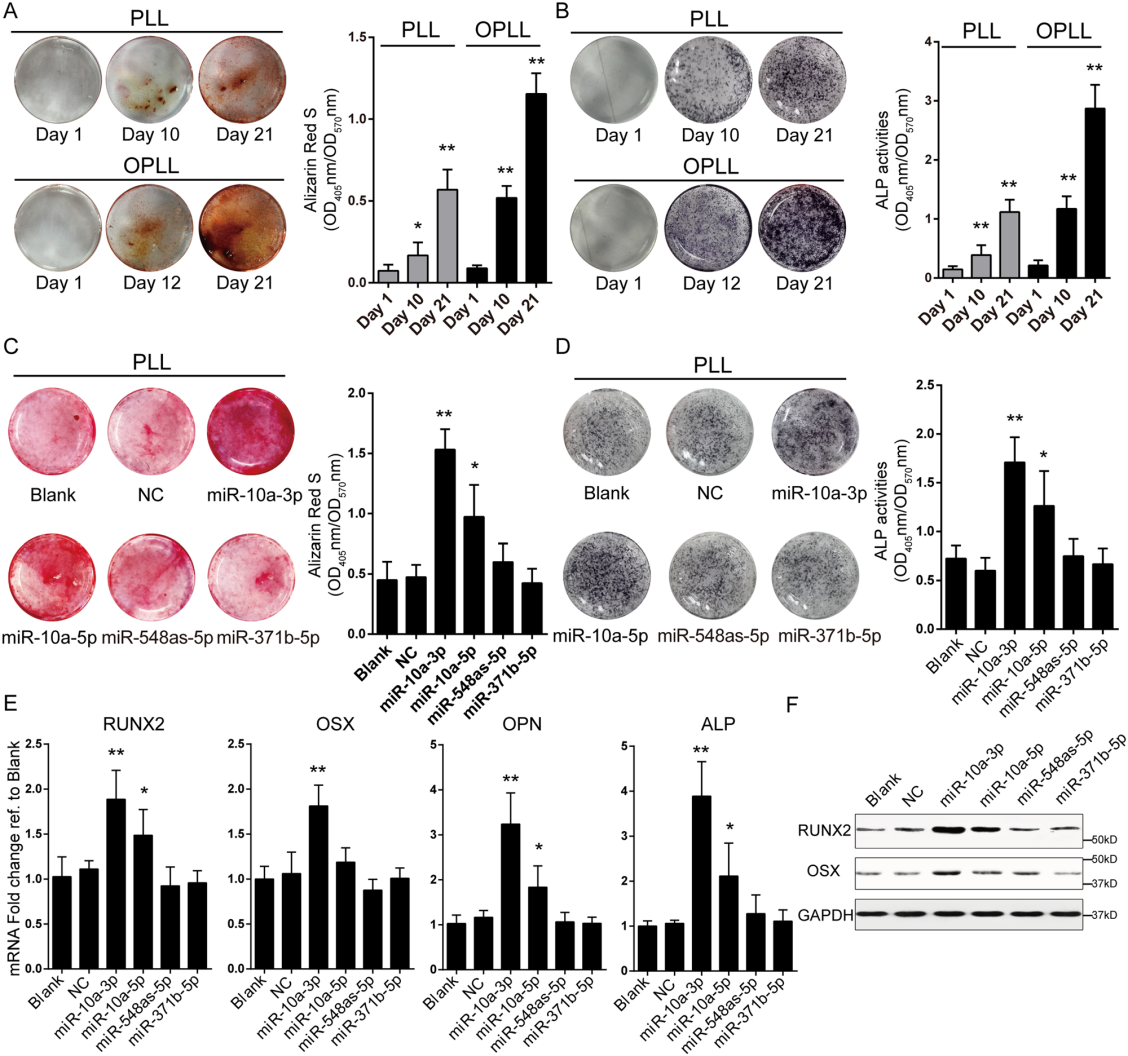
MiR-10a-3p overexpression promotes ossification of posterior ligament cells. The osteogenic properties of PLL and OPLL ligament cells were analyzed using Alizarin Red staining (**A**) or alkaline phosphatase staining (**B**) at different time points of osteo-induction. The colorimetric quantification were shown in the right panels. *P < 0.05, **P < 0.01, n = 3, t-test. The effect of miRNAs overexpression on osteogenic properties of PLL ligament cells were analyzed using Alizarin Red staining (**C**) or alkaline phosphatase staining (**D**) at 12 days after osteo-induction. The colorimetric quantification were shown in the right panels. *P < 0.05, **P < 0.01, n = 3, t-test. (**E**) Real-time PCR analysis of ossification related genes in each treatment group (n = 3) of induced PLL ligament cells. Blank represents osteo-induced cells with no treatment, while NC represents overexpression of a scramble Agomir served as negative control. *P < 0.05, **P < 0.01, t-test. (**F**) Western blot analysis of ossification related genes in each treatment group (n = 2), uncropped images were shown in the Supplementary Fig. 5. The error bars represents ± S.D.

Although the differences between OPLL and PLL cells were previously characterized^10,11^, it is important to confirm that the cell lines we established indeed share common traits with previous reports. We analyzed differences in mineral deposition using alizarin red staining and a quantification method and found that OPLL cells showed more intense staining than PLL cells at 12 days after osteo-induction (Fig. 2A). The colorimetric quantification tests showed almost two times more mineralization of OPLL cells than that of PLL cells at day 21 (Fig. 2A). Similar results were found in the alkaline phosphatase (ALP) assay, in which OPLL cells showed more robust ALP activities than PLL cells (Fig. 2B). These findings were consistent with previous results^11^.

Next, we validated the function of candidate miRNAs using the same approach. By overexpressing the candidate miRNAs, we found that both miR-10a-3p and -5p showed significant increases in alizarin red staining and ALP activities of osteo-induced PLL cells (Fig. 2C,D). The ossification-promoting effects of miR-10a-3p and -5p were further supported by real-time PCR expression analyses of ossification-related *RUNX2*, *OSX*, *OPN* and *ALP* (Fig. 2E). Among these genes, miR-10a-3p overexpression was most effective in elevating the expression level of ossification-related genes. Western blot analyses of RUNX2 and OSX also showed similar changes (Fig. 2F).

**Figure 3:**
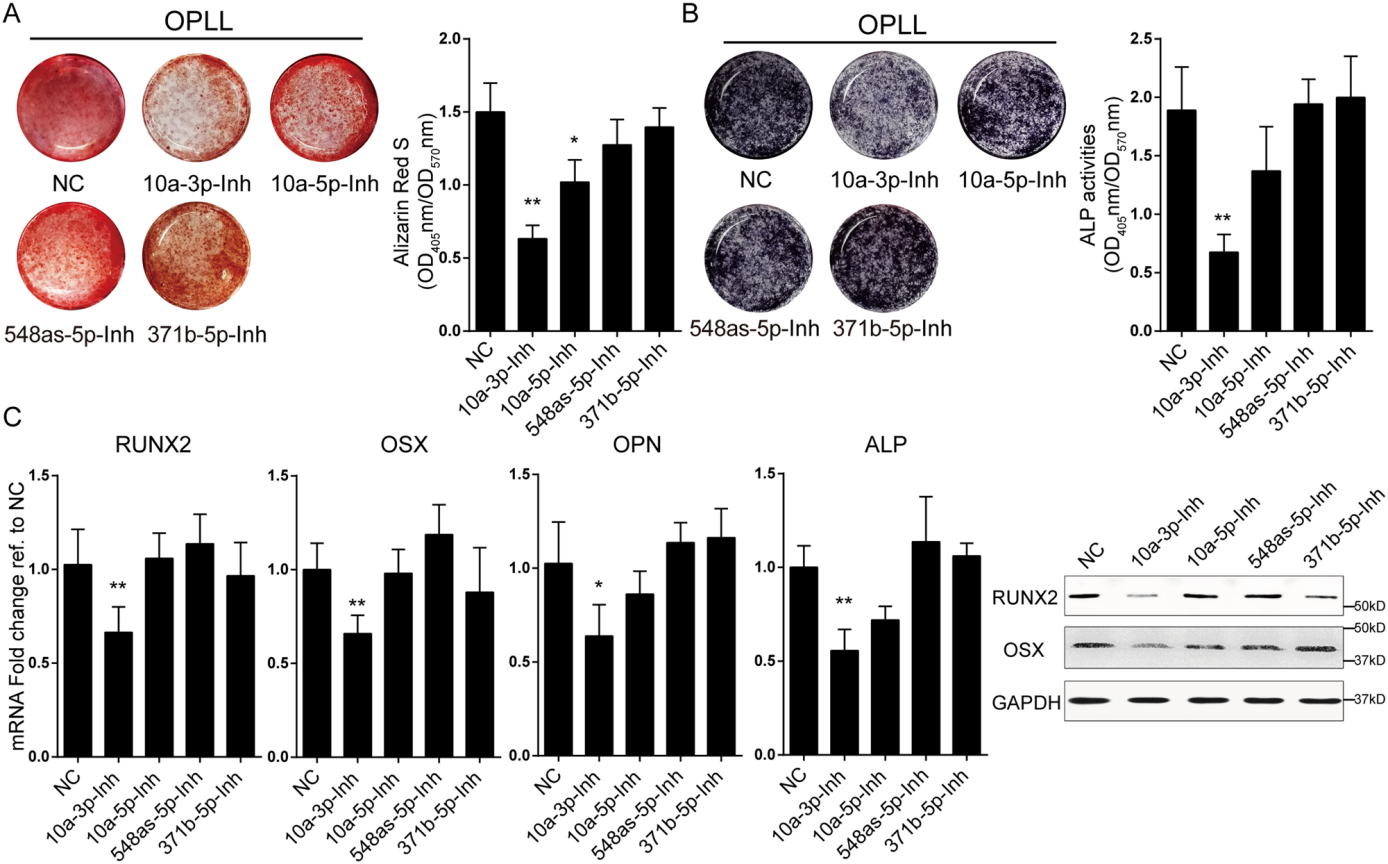
Inhibition of miR-10a-3p reduced osteogenic phenotype of OPLL cells. The effect of miRNAs inhibition on osteogenic properties of OPLL ligament cells were analyzed using Alizarin Red staining (**A**) or alkaline phosphatase staining (**B**) at 12 days after osteo-induction. The colorimetric quantification were shown in the right panels. *P < 0.05, **P < 0.01, n = 3, t-test. (**C**) Real-time PCR analysis of ossification related genes in each treatment group (n = 3) of induced OPLL ligament cells. Blank represents osteo-induced cells with no treatment, while NC represents overexpression of a scramble Antagomir served as negative control. *P < 0.05, **P < 0.01, t-test. (**D**) Western blot analysis of ossification related gene products in each treatment group (n = 2), uncropped images were shown in the Supplementary Fig. 5. The error bars represents ± S.D.

Because of the high expression of these miRNAs in OPLL cells, we designed antagomirs to inhibit their functions in OPLL. We analyzed antagomir-treated OPLL cells using alizarin red staining and ALP activity assays and showed that inhibition of miR-10a-3p, but not miR-10a-5p, significantly reduced the mineral deposition and ALP activities (Fig. 3A,B). Similar results were obtained from analysis of the expression levels of *RUNX2*, *OSX*, *OPN* and *ALP*, which were significantly reduced by miR-10a-3p inhibition at both the mRNA and protein levels (Fig. 3C,D). Taken together, we found that miR-10a-3p can actively regulate the ossification of both PLL and OPLL cells *in vitro*.

### ID3 is targeted by microRNA-10a

**Figure 4:**
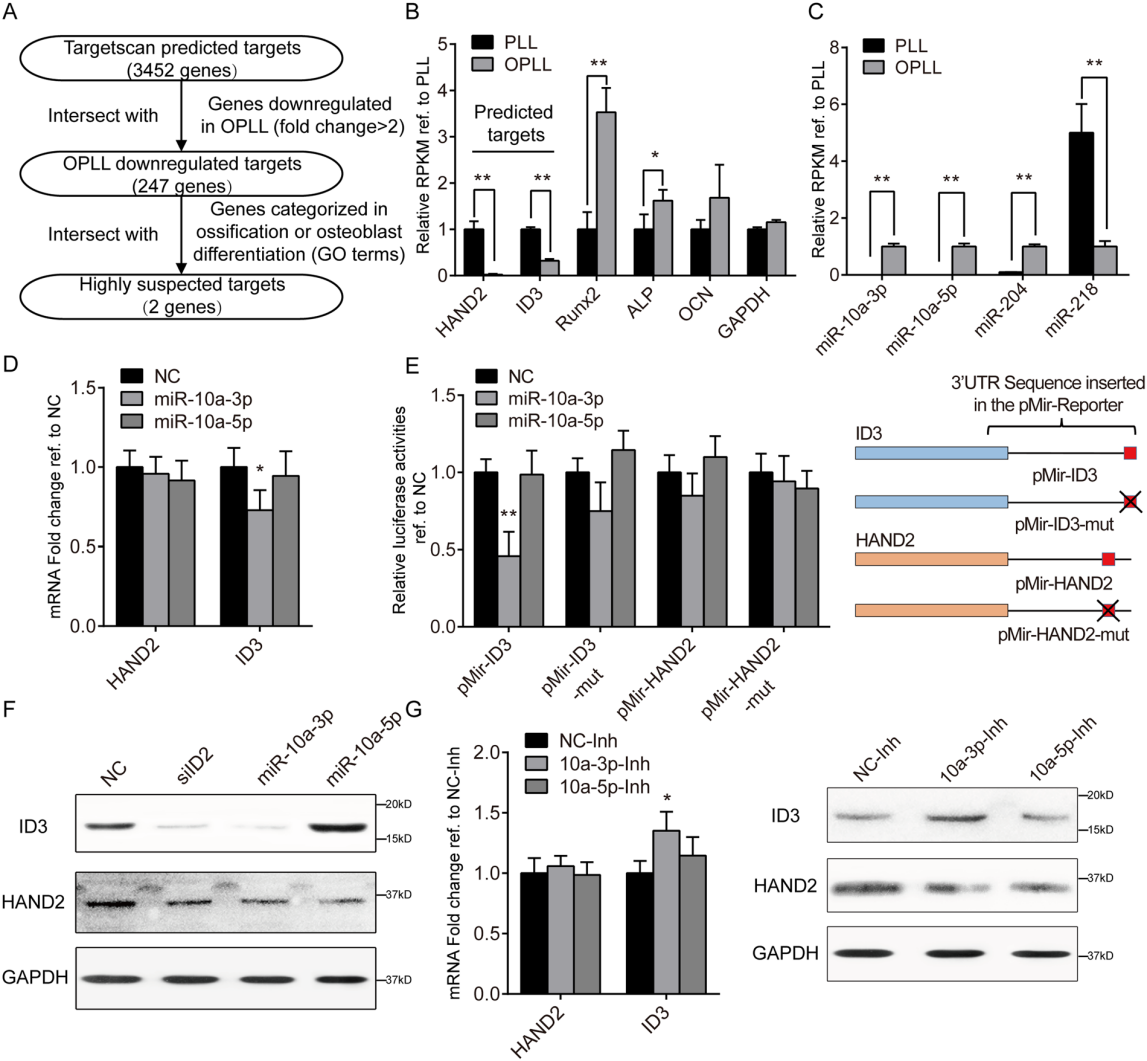
ID3 is targeted by miR-10a-3p. (**A**) A scheme of prediction of potential miR-10a-3p targets. HAND2 and ID3 were the two remaining highly suspected targets of miR-10a-3p used in further analysis. (**B**) Relative RPKM values of the targets and ossification related genes. *P < 0.05, **P < 0.01, n = 3, t-test. (**C**) Relative Transcripts Per Million (TPM) values of miR-10a-3p and other identified ossification related miRNAs. *P < 0.05, **P < 0.01, n = 3, t-test. (**D**) Real-time PCR analysis showing the effect of miR-10a-3p on the RNA level of candidate targets. NC represents overexpression of a scramble Agomir served as negative control. *P < 0.05, n = 3, t-test. (**E**) Dual luciferase reporter assay of wildtype and mutated (-mut) candidate 3′UTR reporters under miR-10a-3p overexpression. The red squares represent predicted miR-10a-3p target sites, while crossed red square represents mutated target sites. Data were normalized to Renilla luciferase activities and compared to the NC group. **P < 0.01, n = 6, t-test. (**F**) Western blot analysis of candidate gene products in each treatment group (n = 2), uncropped images were shown in the Supplementary Fig. 5. (**G**) The effect of miR-10a-3p inhibition on the RNA (left panel) and protein level (right panel) of candidate genes. *P < 0.05, n = 3, t-test. The error bars represents ± S.D.

To identify the relevant targets of miR-10a-3p, we first narrowed down the possible candidates. We filtered the TargetScan predicted targets according to the expression fold change in GSE69787 datasets and further filtered these genes by matching them to the Gene Ontology (GO) term “Ossification” or “Osteoblast differentiation” (Fig. 4A, Supplementary Data 3). After the analysis, *HAND2* and *ID3* were selected as potential targets of miR-10a-3p. We found that the expression changes of these two negative regulators of ossification were indeed inversely correlated with the ossification-related factors RUNX2, ALP and OCN between OPLL and PLL cells (Fig. 4B), while miR-10a-3p was also inversely correlated with the candidates (Figs 4C and S1A–C). These results indicate a connection between miR-10a-3p and the two candidate genes.

To verify this connection, we first tested whether miR-10a-3p could reduce the expression of these two candidates directly. We found that overexpression of miR-10a-3p in PLL cells did reduce the mRNA level of ID3, indicating ID3 may be the effective target of miR-10a-3p (Fig. 4D). Therefore, we further performed dual luciferase reporter assays to verify its effect on protein translation. We found that the luciferase activities from constructs containing the wild type ID3 3′ untranslated transcript region (3′UTR) were significantly reduced after miR-10a-3p overexpression, while mutation in the predicted binding site rescued this reduction (Fig. 4E). Western blot analysis also confirmed the miR-10a-3p-mediated inhibition of ID3 translation, but the effect on HAND2 expression was less notable (Fig. 4F). Next, we investigated whether inhibition of miR-10a-3p using an antagomir would increases the expression of ID3 and HAND2 in OPLL cells. As expected, inhibition using the miR-10a-3p antagomir significantly increased the protein and mRNA level of ID3 (Fig. 4G). Taken together, the results indicated that a negative ossification regulator, ID3, is a functional target of miR-10a-3p in posterior ligament cells.

### The function of RUNX2 is regulated by microRNA-10a

**Figure 5:**
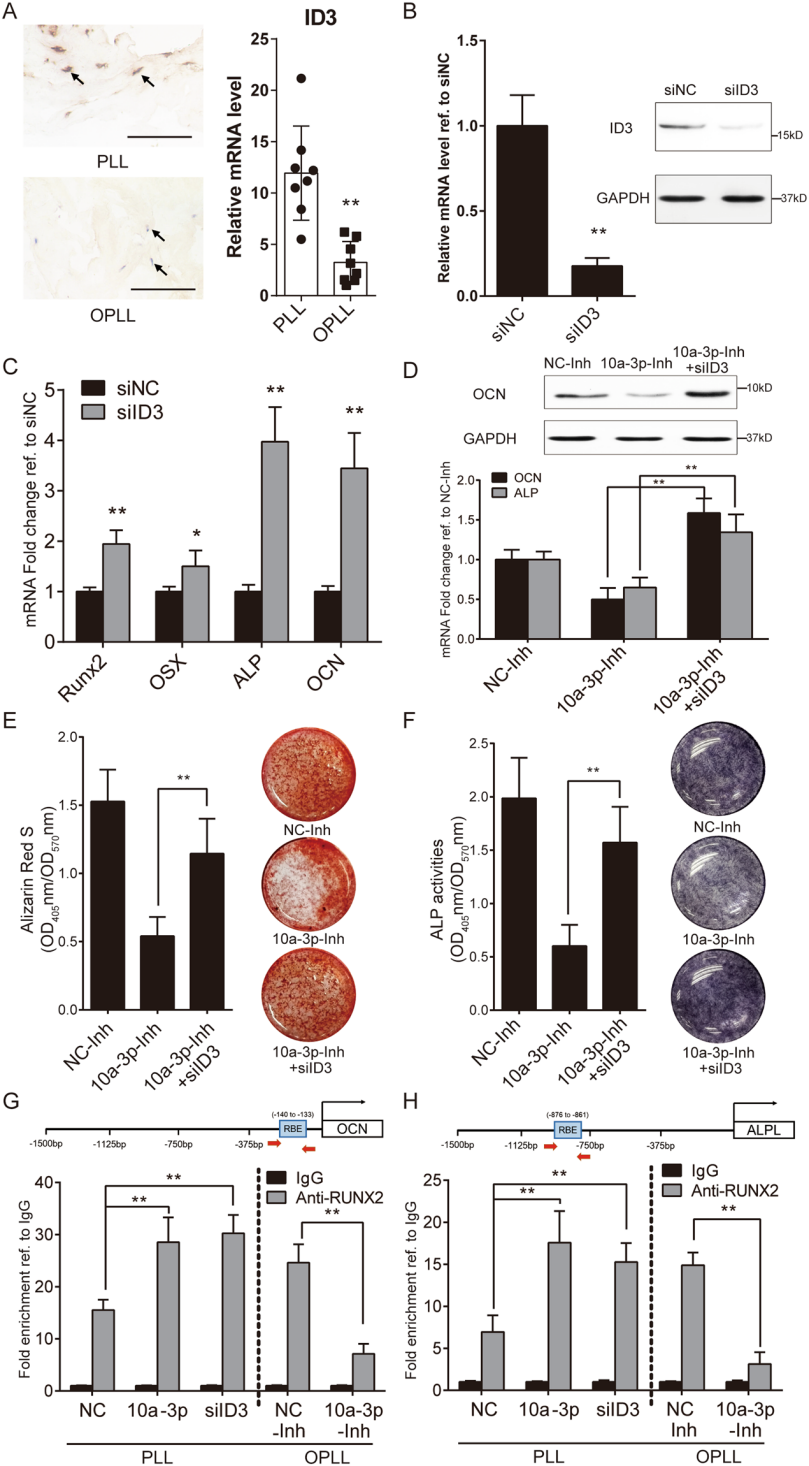
The function of RUNX2 is regulated by miR-10a-3p. (**A**) Evaluation of ID3 expression between PLL and OPLL tissue samples using immuno-histological analysis (left panel, n = 8, scale bars represents 500 μm) and real-time PCR (right panel, n = 8). **P < 0.01, t-test. (**B**) Evaluation of the effect of ID3 knockdown siRNA on ID3 expression. siNC represents scramble single strand siRNA served as negative control. **P < 0.01, n = 3, t-test. (**C**) Real-time PCR analysis showing the effect of ID3 knockdown on ossification related genes. *P < 0.05, **P < 0.01, n = 3, t-test. (**D**) ALP and OCN expression changes during ID3 knockdown on miR-10a-3p inhibited ligament cells. **P < 0.01, n = 3, t-test. Evaluation of the osteogenic phenotypes of ID3 knockdown on miR-10a-3p inhibited ligament cells using alizarin red staining/quantification (**E**) and ALP staining/quantification (**F**). Chromatin immunoprecipitation analysis evaluating the binding proficiency of RUNX2 to OCN (**G**) and ALP (**H**) in miR-10a-3p overexpressed inhibited or ID3 knockdown ligament cells. Data were shown as fold enrichment compared to IgG group. **P < 0.01, n = 3, t-test. The error bars represents ±S.D. Uncropped blot images were shown in the Supplementary Fig. 5.

Although we identified ID3 as the primary target of miR-10a-3p, their relationship in the ossification process of OPLL is unclear. Thus, we first examined whether ID3 expression varies significantly in tissue samples. Immunohistochemistry examination of OPLL (n = 8) and PLL (n = 8) tissue samples showed that the expression of ID3 differed substantially between OPLL and PLL, while real-time PCR analysis of the tissue samples yielded similar results (Fig. 5A). To investigate their relationship and consequences, we introduced an ID3 small interference RNA oligo with 2′ O-Methyl modification and tested its efficiency (Fig. 5B). We showed that ID3 knockdown indeed upregulated the expression of ossification-related genes, which was consistent with a previous report^12^ (Fig. 5C). Next, we performed ID3 knockdown in miR-10a-3p-inhibited osteo-induced OPLL cells and evaluated the ossification phenotype. We found that although miR-10a-3p inhibition attenuated the expression of ossification-related genes, lowered the ALP activities and reduced mineral deposition, ID3 knockdown significantly rescued these changes (Fig. 5D–F). These results indicated that miR-10a-3p-mediated promotion of ossification in posterior ligament cells requires ID3.

How downregulation of ID3 by miR-10a-3p affects ossification-related gene expression is still unknown. Because ID3 is an inhibitor of RUNX2^12^, we performed a chromatin immunoprecipitation assay using an anti-RUNX2 antibody. We analyzed two reported RUNX2 downstream targets, *ALP* and *OCN*^13,14^ in both PLL and OPLL induced cells. After miR-10a-3p overexpression, RUNX2 binding to *OCN* and *ALP* significantly increased in PLL cells, which is similar to the result of ID3 knockdown. While miR-10a-3p inhibition showed the reverse effect in OPLL cells (Figs 5G,H and S2A,B). Thus, we validated the function and mechanism of miR-10a-3p in PLL and OPLL primary ligament cells *in vitro*.

### MicroRNA-10a promotes ossification by targeting ID2/RUNX2 signaling *in vivo*

**Figure 6:**
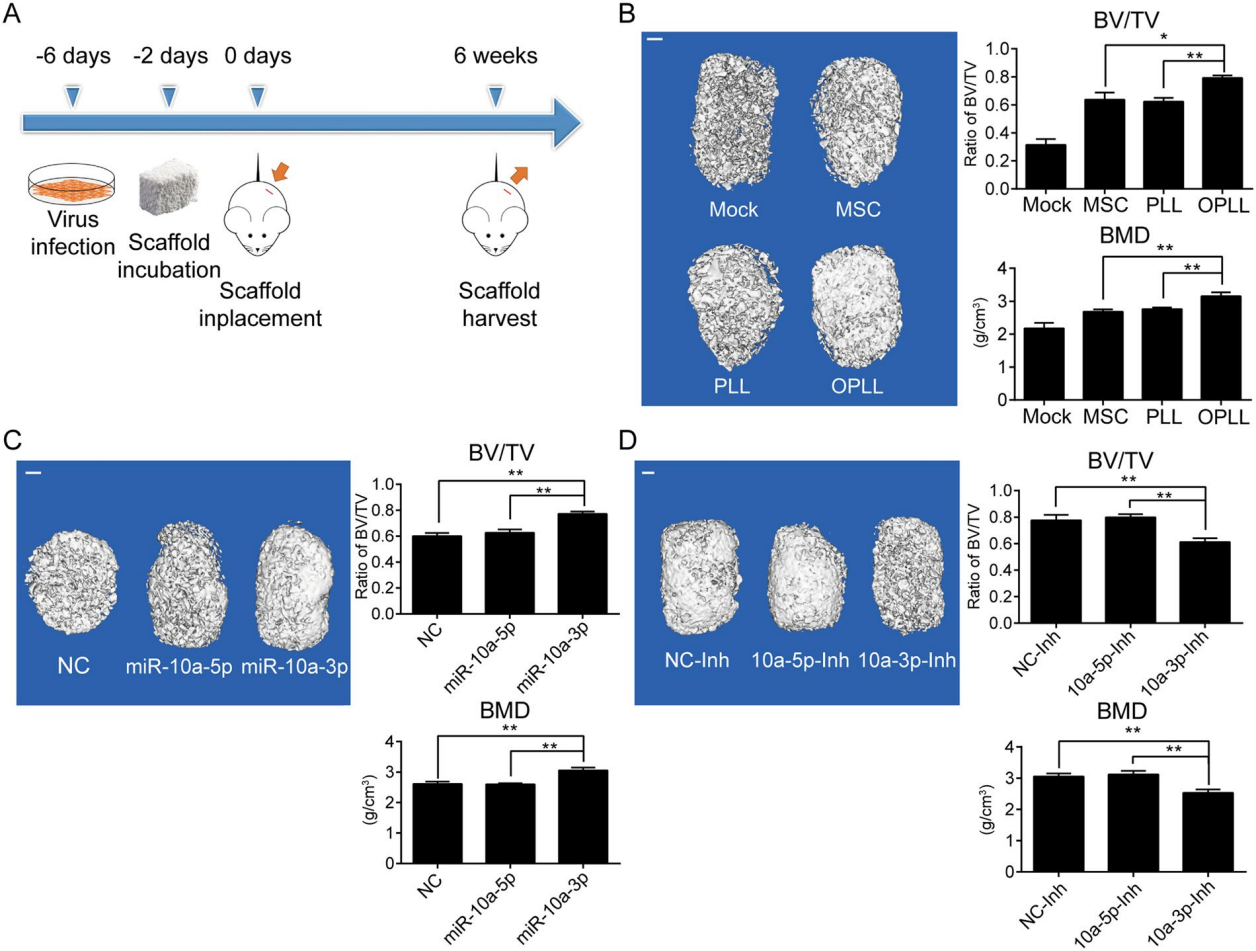
MiR-10a-3p promotes the ossification of OPLL *in vivo*. (**A**) A scheme of the procedure in heterotopic bone formation assay. (**B**) Reconstructed 3D micro-CT images of the tissue-engineered bone constructs from different type of cells seeded (n = 6). The ratio of bone volume to tissue volume (BV/TV) and bone mineral density (BMD) of cultured bone constructs were shown in the right panel. *P < 0.05, **P < 0.01, t-test. (**C**) Reconstructed 3D micro-CT images of the tissue-engineered bone constructs that contains miR-10a-3p, miR-10a-5p or negative control stably expressed PLL cells (n = 6). The ratio of bone volume to tissue volume (BV/TV) and bone mineral density (BMD) of cultured bone constructs were shown in the right panel. **P < 0.01, t-test. (**D**) Reconstructed 3D micro-CT images of the tissue-engineered bone constructs that contains miR-10a-3p, miR-10a-5p or negative control stably inhibited OPLL cells (n = 6). The ratio of bone volume to tissue volume (BV/TV) and bone mineral density (BMD) of cultured bone constructs were shown in the right panel. **P < 0.01, t-test. The error bars represents ±S.D.

To fully characterize the function of miR-10a-3p in OPLL development, we used an *in vivo* bone formation strategy. PLL cells stably expressing miR-10a-3p or OPLL cells stably expressing miR-10a-3p short hairpin inhibitor (Fig. S3A,B) were incubated with Bio-Oss Collagen scaffolds and implanted subcutaneously, and the scaffolds were harvested after 6 weeks and analyzed using micro computed tomography (micro-CT, Fig. 6A). Here, we first evaluated the bone formation ability of different cell types. The results showed that the ratio of bone volume/tissue volume (BV/TV) and bone mineral density (BMD) were significantly increased in the OPLL group compared to the PLL group and mesenchymal stem cell (MSC) group, which served as a positive control because it has been previously studied in this model^15^ (Fig. 6B). In PLL groups (Figs 6C and S3C), stable miR-10a-3p overexpression resulted in increased BV/TV and BMD compared to the negative control group and stable miR-10a-5p overexpression group (which served as a negative miRNA control group). In the OPLL groups (Figs 6D and S3D) stable miR-10a-3p knockdown resulted in lower BV/TV and BMD compared with the other treatment groups. These results further confirmed that miR-10a-3p, not its complementary miR-10a-5p, is functional in promoting posterior ligament cell ossification *in vivo*.

**Figure 7:**
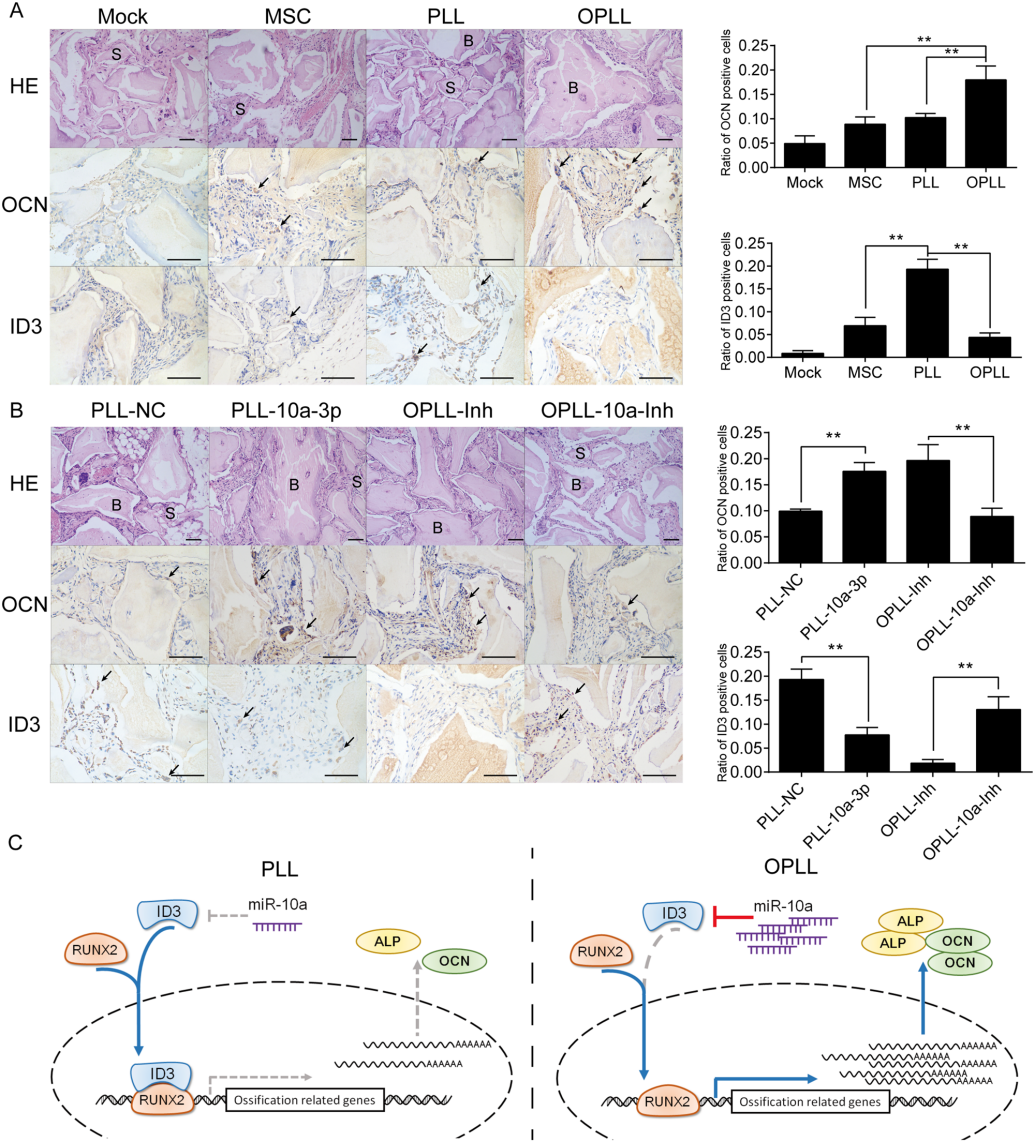
ID3 is modulated by miR-10a-3p *in vivo*. (**A**) HE staining and immunohistological staining of OCN and ID3 in different cell type seeded bone grafts. Note bone formation (**B**) around the degradation scaffold (S) in the region. Representative positively stained cells are marked with black arrows. Scale bar represents 50 μm. Quantification of the ratio of positive cell counts were shown in the right panel. **P < 0.01, n = 6, t-test. (**B**) HE staining and immunohistological staining of OCN and ID3 in different treatment ligament cell seeded bone grafts. Scale bar represents 50 μm. Quantification of the ratio of positive cell counts were shown in the right panel. **P < 0.01, n = 6, t-test. (**C**) Schematic of the miR-10a-3p/ID3/RUNX2 axis in promoting OPLL development. The error bars represents ±S.D.

Histological examination also validated the previous findings *in vivo*. Hematoxylin and eosin (HE) staining showed that bigger lamellar bone with osteocyte lacunae or more organized extracellular matrix was formed in the OPLL group than other cell type groups (Fig. 7A). However, miR-10a-3p overexpression resulted in increased lamellar bone formation, and its inhibition led to decreased bone formation (Fig. 7B). HLA-A staining confirmed that the seeded cells were blended with the scaffold and became the majority population in the scaffold we harvested (Fig. S4A). Immunohistochemistry staining of OCN and RUNX2 confirmed that miR-10a-3p overexpression resulted in more positively stained cells, while its inhibition resulted in fewer stained cells (Figs 7B and S4B). ID3, however, showed more positively stained cells after miR-10a-3p inhibition, while its overexpression resulted in decreased staining (Fig. 7B).

Thus, we confirmed the function and mechanism of miR-10a-3p in promoting posterior ligament cell ossification both *in vitro* and *in vivo*. Using inhibition of miR-10a-3p in OPLL cells and overexpression in PLL cells, we showed that miR-10a-3p can actively modulate the ossification level of ligament cells by targeting ID3, thus promoting the binding of RUNX2 to ossification-related genes (Fig. 7C).

## Discussion

Ossification of the posterior longitudinal ligament (OPLL) is a common spinal disease that often causes severe injuries and compressions to the spinal cord and nerve roots. OPLL is characterized by ectopic ossification in the posterior ligament of the spine, patients of which could develop myelopathy, radiculopathy or even quadriplegia^1^. Although OPLL is traditionally believed to be more common in East Asians, with a prevalence of 2 to 4% compared with 0.01 to 2% in North Americans and Europeans^16^, however, recent reports showed increasing incidence of OPLL in Caucasian degenerative cervical myelopathy patients^17,18^, indicating a worldwide problem that requires further attention. Although previous studies have suggested that the causes of OPLL are multifactorial in nature, the underlying mechanism and regulatory networks have not been fully elucidated. Single nucleotide polymorphisms (SNP) and mutations of various genes, including *BMP-2, PTCH1, BMP4, TGF-β1, TGF-β3, COL6A1, NPP1* and *RUNX2*, have been associated with OPLL development, which indicates that genetic factors may play key roles in this process^2–5^. But they have been shown to play vital roles in many ossification-related events and diseases, indicating that they are important “effectors” rather than “activators”.

Recently, various miRNAs that regulate osteogenic differentiation have been identified. For example, miR-690 activated by RUNX2 positively regulates RUNX2-induced osteogenic differentiation by inactivating the NF-κB pathway via the downregulation of subunit p65^7^, whereas TNF-α-induced NF-kB activation upregulates microRNA-150-3p and inhibits osteogenesis of mesenchymal stem cells by targeting β-catenin^8^. These results suggest that osteogenesis is tightly controlled by miRNAs, and miRNA regulation may be an upstream mediator of the osteogenic process. The development of OPLL also involves pathological processes that is similar to osteogenesis, during which specific miRNA regulation and expression changes may also be responsible for the activation or repression of ossification-related factors. A recent study done by our group defined OPLL-specific microRNAs (miRNAs) using high-throughput miRNA sequencing and revealed that their predicted regulatory network was closely related to ossification development^9,19^. Our investigations found that among the four top regulated miRNAs, miR-10a-3p strongly promoted PLL cell ossification, indicating that miR-10a may be a critical regulator of this pathological process. Further functional studies also confirmed the hypothesis that miR-10a-3p actively downregulate the expression of ID3, promoting the function of the ossification core factor RUNX2.

MiR-10a is an evolutionarily conserved microRNA that has been shown to have vital roles in many pathological processes, including those of rheumatoid arthritis^20^, juvenile dermatomyositis^21^, and various types of cancers^22–25^. In our study, we combined *in vitro* osteo-induction examination and *in vivo* bone formation assays to show that miR-10a also plays vital roles in the osteogenesis of posterior ligament cells. However, a recent report indicated that miR-10a has a suppressive role in osteoblast differentiation of MC3T3-E1 cells and angiogenic activity of MUVECs by downregulating β-catenin expression^26^. Notably, β-catenin levels were not significantly varied between PLL and OPLL ligament cells^9^, and the mechanism which Li *et al*. proposed is between miR-10a-5p and β-catenin, not the miR-10a-3p we focused on. Moreover, computational prediction in our study yielded 0 potential ossification-related targets for miR-10a-5p, which indicates that miR-10a-5p may function differently in OPLL via other mechanisms. Nevertheless, the function of miRNAs relies greatly on the miRNA binding target. Reports have shown that although many mRNAs share the same seed region for one miRNA^27^, the primary or functional target that one miRNA may affect depends on the expression level of the target or the miRNA/target ratio^28^. Here, our findings showed that both miR-10a-3p and its target ID3 differs greatly between PLL and OPLL, making it a possible functional pair that take an active role in the development of OPLL.

On the other hand, *Inhibitors of differentiation (ID)* genes are evolutionarily conserved transcriptional regulators involved in multiple cellular processes including cell proliferation, differentiation and ultimately, defining cell fate^29^. The members of *ID* gene family (Id1-Id4) share conserved sequence homology with basic helix-loop-helix motif (bHLH) but lack the basic DNA-binding domain that bind E-box sequences^30^. Thus could form heterodimers with bHLH proteins to prevent the proper functioning of bHLH transcription factors. Moreover, ID proteins determine cellular responses to TGFβ and BMP signaling pathway. Of the four members, ID1 and ID3 expression have been reported to be down-regulated in TGFβ–driven epithelial-mesenchymal transitions (EMT) and up-regulated by BMPs^31,32^, indicating the reverse role of ID proteins and BMP signaling. A Study by Luan *et al*. reported that ID2 functions to inhibit the binding of RUNX2, an osteoblast-specific transcription factor, to its downstream ossification related factors eventually inhibit the osteogenesis process^12^. In the present study, we found that miR-10a-3p exerts its function through the ID3/RUNX2 axis, which defined their upstream modulator and for the first time revealed their vital roles in the development of OPLL.

Our findings indicate that miR-10a-3p is indeed a vital player in osteogenesis of OPLL and serves as an upstream regulator of the ID3/RUNX2 axis. However, one of the main drawbacks of this study is that how miR-10a expression is initiated in OPLL are still unclear and require further investigation^33^. DNA methylation and histone modifications are identified to play critical roles in chromatin remodeling and general regulation of gene expressions in mammalian development and human diseases, such factors can also influence the expression of microRNAs, like cancer^34^. However, the global DNA methylation and histone modifications status of OPLL is not studied which may be important for better understanding of such regional intensive diseases. Another main problem with the studying of OPLL is lack of animal models. The tip-toe walking mice are the most accepted OPLL animal model for its assemblance in the spinal ligament ossification^35^. However, tip-toe walking mice are ENPP mutated, a gene that suppress ossification in all tissues^36^, and such mutation caused it hard to control the exact timing and radius of the ossification. Although there are other transgene animal models claimed to be suitable for OPLL study^37^, lack of verification and evidence held them from further usage.

## Methods

### Sample collection and primary cell culture

The diagnosis of OPLL or PLL (spinal trauma patients who underwent cervical corpectomy) was confirmed by computerized tomography (CT), and magnetic resonance imaging preoperatively in our institution. OPLL or PLL specimens obtained during surgery were immediately put to primary cell culture as previously reported^9^. In brief, the ligaments were rinsed with PBS twice, then carefully dissected under microscope to avoid any contamination with bone or disc tissues. The collected tissues were minced, washed and plated on a 100 mm culture dish with Dulbecco’s Modified Eagle Medium (DMEM, Life technology Gibco, USA) supplemented with 10% fetal bovine serum (FBS, Gibco, USA), 1% L-glutamine (Gibco, USA), and 1% penicillin/streptomycin (Gibco, USA) in humidified atmosphere containing 95% air and 5% CO_2_ at 37 °C. The fibroblast-like cells that migrated were harvested with 0.02% EDTA)/0.05% trypsin and plated in T25 culture flask for further expansion and analysis. The ethics committees of the Changzheng Affiliated Hospital of Second Military Medical University approved the study protocols, and each participant have written informed consent. The methods were carried out in accordance with the approved guidelines. We totally obtained 12 OPLL patient tissue samples during anterior cervical discectomy, and 9 PLL patient sample during anterior cervical corpectomy.

### MicroRNA analysis and target prediction profiling

The differentially expressed miRNAs between PLL and OPLL were identified in our previous study using parameters of P ≤ 0.01 and fold change ≥2 or ≤0.5^9^. Hierarchical clustering was performed as described^38^. We used Cluster 3.0 and R package to re-order differentially expressed miRNAs by fold change. For miRNA prediction, Targetscan (www.targetscan.org) was used to predict the binding of differentially expressed miRNAs to the putative targets. The predicted target genes were compared with the transcriptome profiling data of GSE69787, and only genes that are significant (P ≤ 0.01 and fold change ≥2 or ≤0.5) and inversely correlated in expression with the targeting miRNA were included. Gene Ontology analyses were done by comparing the enrichment of each GO term using the DAVID (david-d.ncifcrf.gov).

### Dual luciferase assay and oligonucleotide syntheses

For dual luciferase report assay, the Reporter constructions were performed by synthesizing wild type or mutated 3′UTR of ID3 and HAND2, and subcloned into pMir-report vector (Promega, WA, USA), which all these procedures were done by Obio Technology Corp (Shanghai, China). ligament cells were plated in 96-well plates and transfected using Lipofectamine 2000 (Invitrogen) with 50 ng pMir-report vector (carrying firefly luciferase) with indicated 3′UTR of wild type or mutated ID3 or HAND2. Empty vector served as a control, and a PRL-TK vector (carrying Renilla luciferase) served as internal control (Promega, WA, USA).

For miRNA overexpression and inhibition, miRNA Agomirs and Antagomirs were synthesized by GenePharma Corp (Shanghai, China). Agomirs and Antagomirs are all specially labeled and chemically-modified RNA oligonucleotides, designed based on the mature microRNA sequence. A scramble miRNA mimic control was used as negative control. For ID3 silencing, two validated siRNAs were designed and synthesized by GenePharma Corp with 2′-Ome modification, and were combined to achieve better silencing effect. A scramble siRNA control that target none of the IDs was used as negative control. The indicated Agomirs, Antagomirs, siRNAs or scramble controls were transfected at a final concentration of 20 pmol/ml.

### Real-time PCR

Total RNA were extracted by TRIzol solution (Invitrogen, Carlsbad, USA) and reverse transcribed using ReverTra Ace® qPCR RT Kit (Toyobo, Osaka, Japan). Single strand cDNA were analyzed with SYBR Green master mix (Roche, USA) according to the instructions made by the manufacturer. We used StepOne Plus system (Thermo Fisher Scientific, New Jersey, USA) to perform the qPCR analysis, and a 2^−ΔΔCT^ method was used for calculating the fold changes in each experiments. Primers used in the real-time PCR were listed in the Supplementary Data 4.

### Western Blot

Western blot analysis was performed as described previously^28^. Primary antibodies against RUNX2 (20700-1-AP, ProteinTech, Wuhan, China), OSX (ab22552, Abcam, Beverly, USA), ID3 (ab41834, Abcam, Beverly, USA), HAND2 (ab56590, Abcam, Beverly, USA), and GAPDH (ProteinTech, Wuhan, Shanghai) were used in the analysis at the dilution ratio of 1:1,000.

### Alizarin Red, Alkaline phosphatase staining and quantification

Before the analysis of osteogenic properties, cells were treated with osteogenic induction medium consisting of DMEM with 10% FBS, 25 mg/ml ascorbate-2 phosphate, 10^−8^ M dexamethasone, and 5 mM β-glycerophosphate (All from Gibco, USA) for the indicated time (otherwise 2 weeks). After induction, cells were fixed with 4% paraformaldehyde, after few washes with PBS, cells were stained according to the manufacturer’s instruction. We used Alizarin Red S Staining Kit (#0223, Sciencell, San Diego, USA) for calcium deposition analysis, Alizarin Red S Staining Quantification Assay kit (#8678, Sciencell, San Diego, USA) was used for quantification. Alkaline phosphatase staining kit (1102-100, Sidansai, Shanghai, China) was used for cellular ALP staining, and Alkaline Phosphatase Assay kit (ab83369, Abcam, Beverly, USA) for quantification. The average value of OD_405nm_ of each sample were calculated, and the average value of OD_570nm_ of each sample were also calculated as background control. Values of OD_405nm_ were divided by OD_570nm_ to generate a standardized OD value for each sample for further comparison. Infinite™ M200 (Tecan, USA) microplate plate reader was used for absorbance quantification, and three replicates were done to generate the average value.

### ***In vivo*** heterotopic bone formation assay

MiR-10a-3p overexpression and short-hairpin silencing lentivirus vectors were all synthesized and cloned by Obio Technology Corp (Shanghai, China). The PLL, OPLL or MSC cells were first passaged and when 80% confluent, the cells were treated with indicated virus vectors to modify their expression of miR-10a-3p. Three days after treatment, the cells were selected using puromycin and the cells were resuspended the next day to incubate with 7 mm × 5 mm × 2 mm Bio-Oss Collagen (Geistlich, GEWO GmbH, Baden, Germany) scaffold. At the same time of incubation, the medium were changed to osteogenic medium to induce osteogenic differentiation of the cells. Two days after the incubation, cell seeded scaffolds were implanted subcutaneously on the back of 4-week-old BALB/c homozygous nude (nu/nu) mice (6 mice per group), as described previously^15^. Implants were harvested 6 weeks after the transplantation and fixed in 4% paraformaldehyde. All mice were purchased from Shanghai SLAC Laboratory Animal Co. Ltd (Shanghai, China), and were individually caged under standard conditions (12 hours light/12 hours dark cycle, 24 °C controlled temperature). All animal experiments were approved by the Second Military Medical University Animal Care and Use Committee and fulfill the ARRIVE Guideline Requirements.

### Micro-CT analysis

Micro-CT analysis of specimens was performed using a high-resolution Inveon Micro-CT (Siemens, Munich, Germany) provided by Shanghai Model Organisms Center Inc. The 3D reconstruction and volume quantification of the ectopic bone were performed using standardized thresholds as described previously^15^. The region of interest was selected (2 × 2 × 1 mm in size), and the lower and upper threshold values for bone were set. Relevant parameters, including bone mineral density (BMD, mg/cc) and the ratio of new bone volume to existing tissue volume (BV/TV) were analyzed and calculated.

### H&E staining and immunohistochemical analysis

The specimens were decalcified in 10% ethylene diamine tetraacetic acid (EDTA, pH 7.4) for 1 month, followed by dehydration and embedding in paraffin. Sections (5 μm) were cut and stained with hematoxylin and eosin (HE). Meanwhile, sections were evaluated by immunohistochemical analysis, as described previously [26]. The specimens were blocked with 3% BSA for 30 min and then incubated with primary antibody against OCN (23418-1-AP, ProteinTech, Wuhan, China), RUNX2 (20700-1-AP, ProteinTech, Wuhan, China), HLA (ab52455, Abcam, Beverly, USA) and ID3 (ab41834, Abcam, Beverly, USA) at 4 °C overnight. Then, sections were processed using the ABC detection kit (Vector Laboratories, Burlingame, CA) and visualized under an Olympus BX51 light microscope equipped with Olympus DP70 camera (Olympus Co., Tokyo, Japan).

### Chromatin immunoprecipitation analysis

ChIP assays were performed in accordance with the manufacturer’s instructions of the EZ-Magna ChIP A/G Kit (Millipore, Billerica, MA, USA). ALPL (11187-1-AP, ProteinTech, Wuhan, China) and RUNX2 (20700-1-AP, ProteinTech, Wuhan, China) antibodies were used in the analysis, an anti-rabbit IgG antibody were used as negative control.

### Statistical analysis

Data are reported as mean values ± SD (standard derivation). Data analysis was performed using SPSS for Windows version 16.0. Student’s t test and one-way ANOVA were performed where appropriate and a *P* value < 0.05 was considered as statistically significant.

## Electronic supplementary material

Supplementary Information

Supplementary Data 1

Supplementary Data 2

Supplementary Data 3

Supplementary Data 4
