# Supplementary material for: The microRNA-10a/ID3/RUNX2 axis modulates the development of Ossification of Posterior Longitudinal Ligament
Source: Sci Rep. 2018 Jun 15;8:9225. doi: 10.1038/s41598-018-27514-x (PMC6003989; doi:10.1038/s41598-018-27514-x)
Supplement: Supplementary file 1 — Supplementary Information [file 41598_2018_27514_MOESM1_ESM.pdf]

Supplementary Information for

The microRNA-10a/ID3/RUNX2 axis modulates the development of Ossification of Posterior Longitudinal Ligament

Authors: Chen Xu[1,*], Hao Zhang[1,*], Wei Gu[1,*], Huiqiao Wu[1,*], Yuanyuan Chen[1,2], Wenchao Zhou[1], Baifeng Sun[1], Xiaolong Shen[1], Zicheng Zhang[4], Yue Wang[3,5], Yang Liu[1,5] and Wen Yuan[1,5]

Affiliations: [1] Spine Center, Department of Orthopaedics, Changzheng Hospital, Second Military Medical University, 415th Feng Yang Road, Shanghai, 200003, PR China.

[2] Department of Orthopedic Surgery, Sixth People's Hospital Affiliated to Shanghai Jiao Tong University, 800th Yi Shan Road, Shanghai, 200233, PR China.

[3] Research Center of Developmental Biology, Second Military Medical University, 800th Xiang Yin Road, Shanghai, 200433, PR China.

[4] Administration Office for Graduate Students, Changhai Hospital, Second Military Medical University, 168th Chang Hai Road, Shanghai, 200433,PR China.

[5] Corresponding authors, Prof. Yang Liu is the lead corresponding author.

[*] These authors contributed equally to this work.

Supplementary Information Include

Supplementary figures and legends: Figure. S1-S4

Supplementary Dataset 1 Differentially expressed miRNAs in OPLL

Supplementary Dataset 2 GO analysis of miRNA's predicted targets

Supplementary Dataset 3 Predicted targets and their GO categories

Supplementary Dataset 4 Oligonucleotide Sequences used in this study.

Supplementary figures and legends

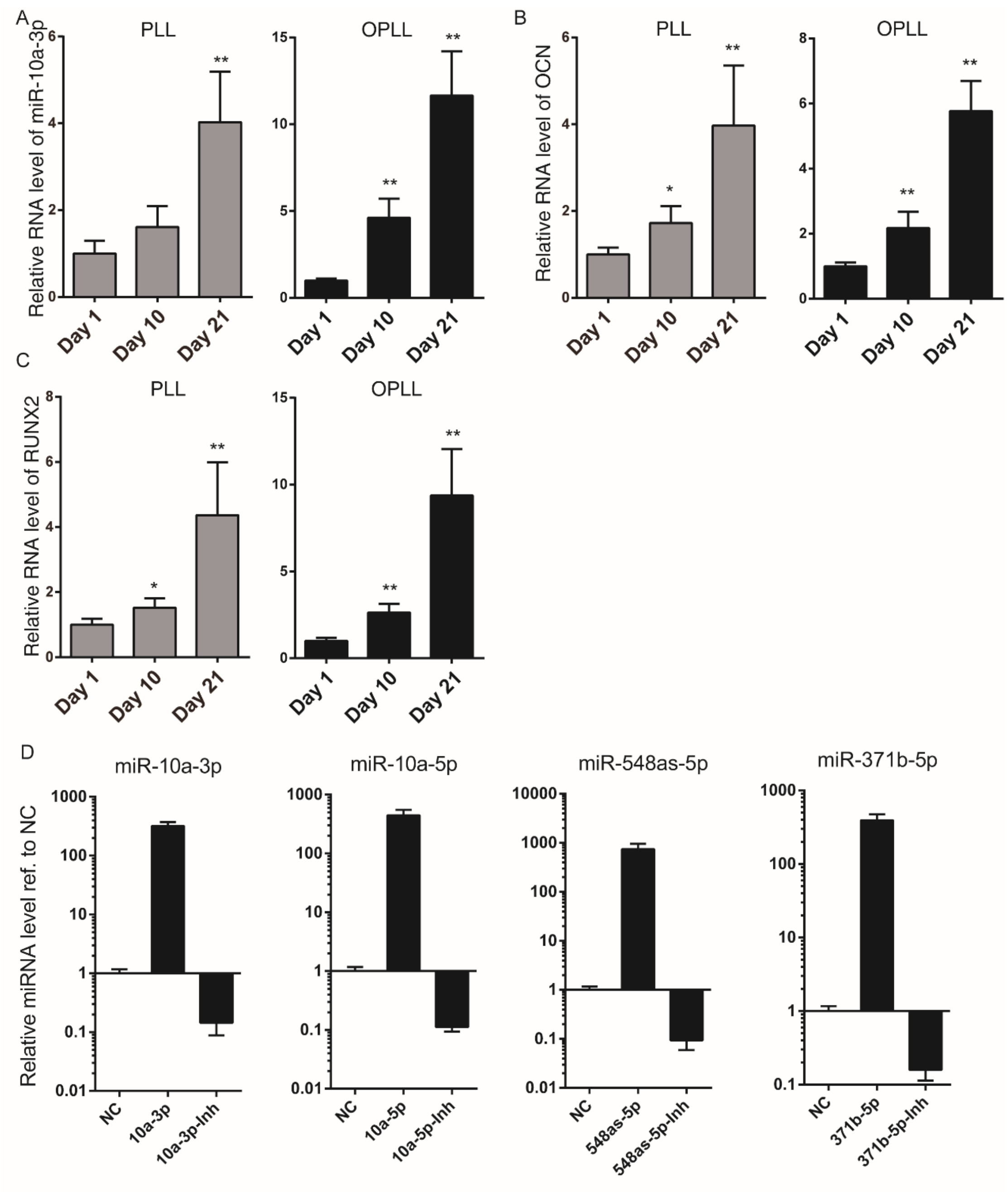

Figure. S1. Real-time PCR analysis of miR-10a-3p (**A**) and ossification related OCN (**B**), RUNX2 (**C**) in each treatment group (n=3) of induced PLL or OPLL ligament cells at each time point. Real-time PCR validations (n=3) of microRNA agomir overexpression and antagomir inhibition in the ligament cells respectively (**D**). **P*<0.05, ***P*<0.01, t-test.

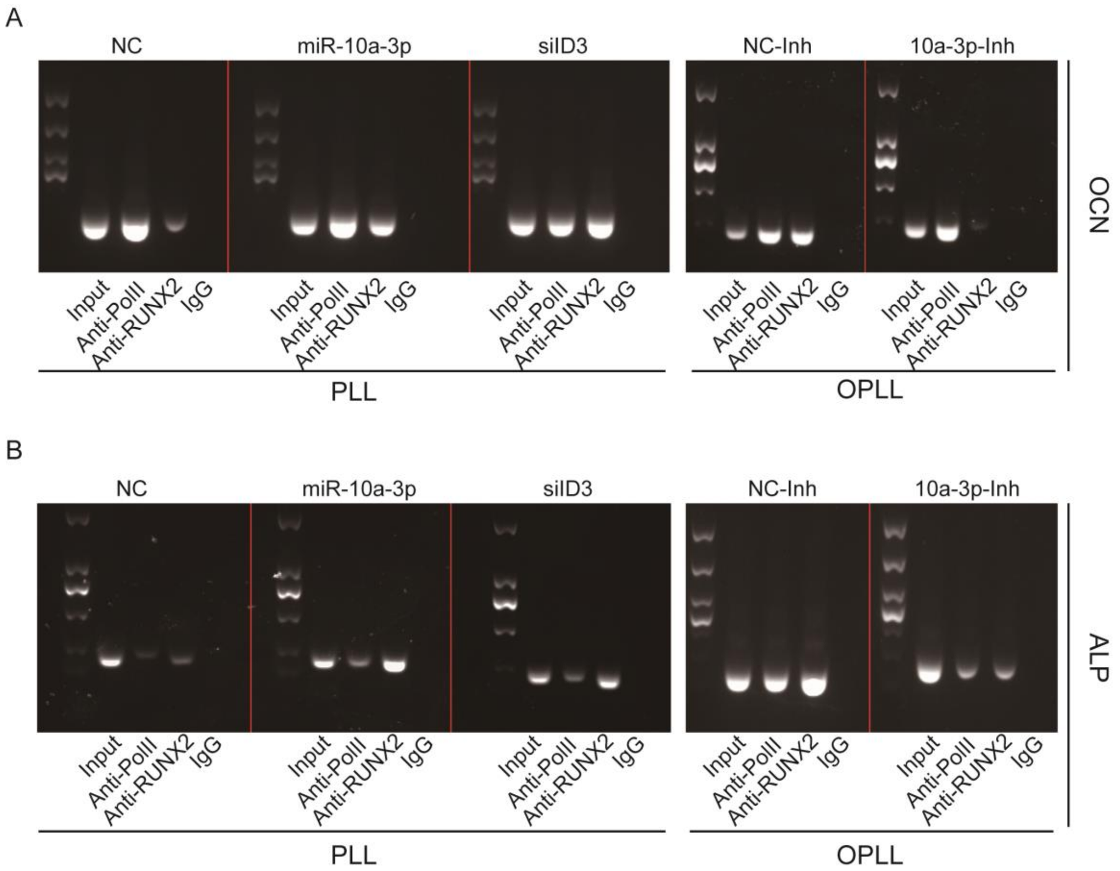

Figure. S2. Chromatin immunoprecipitation analysis evaluating the binding proficiency of RUNX2 to OCN **(A)** and ALP **(B)** in miR-10a-3p overexpressed or inhibited ligament cells. Representative images of the electrophoresis of the PCR products were shown.

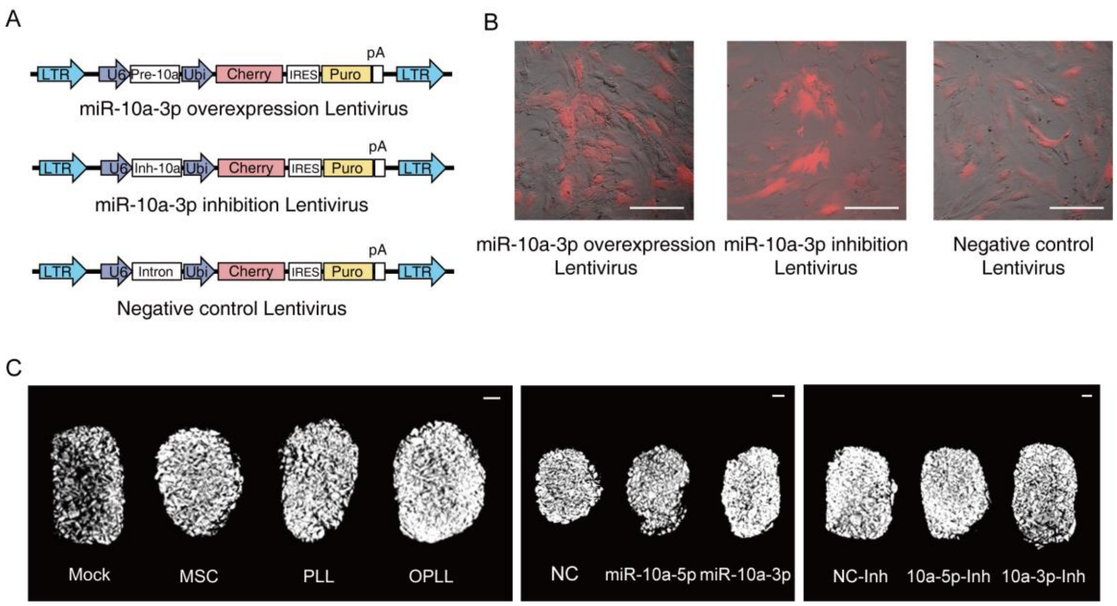

Figure. S3. Constructed lentivirus vectors **(A)** and the infected ligaments cells express strong Cherry fluorescence observed **(B)**. Representative micro-CT images **(C)** showing tissue-engineered bone constructs from different type of cells (left panel), miR-10a-3p, miR-10a-5p or negative control stably expressed PLL cells and miR-10a-3p (middle panel), miR-10a-5p or negative control stably inhibited OPLL cells (right panel).

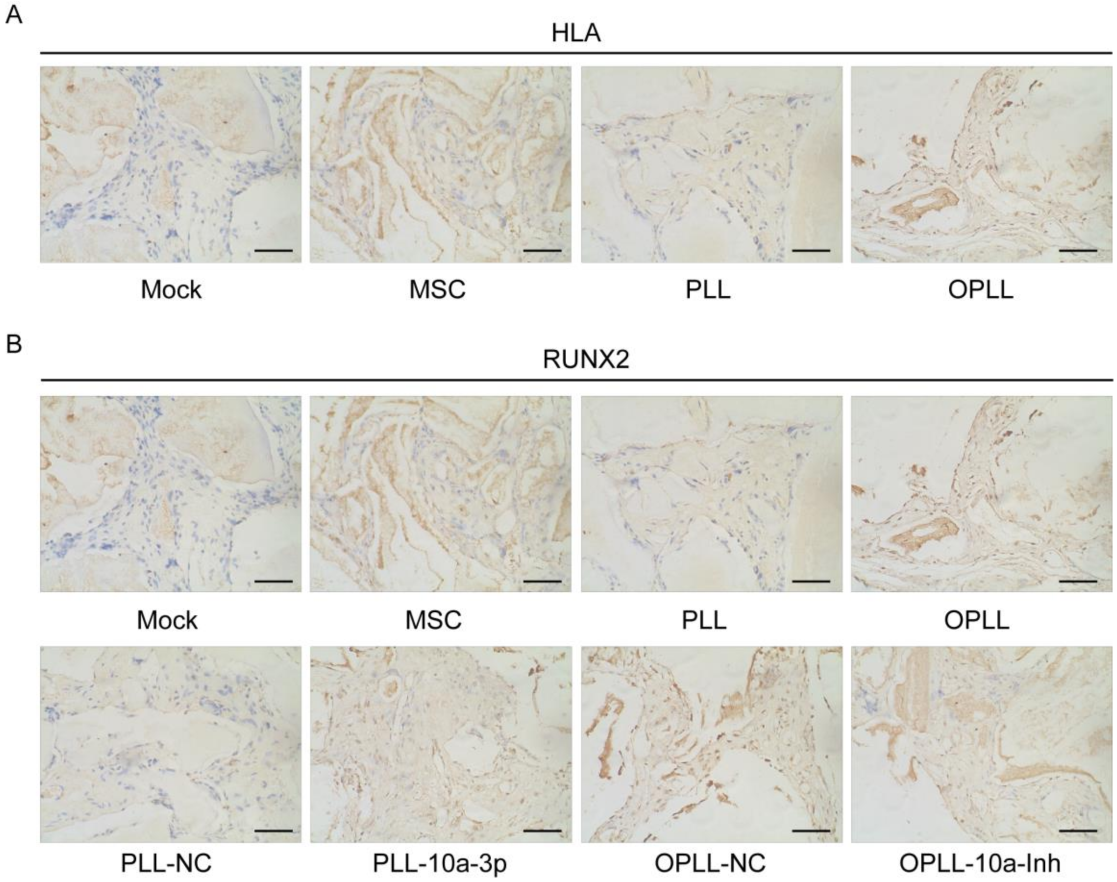

Figure. S4. Immunohistological staining of HLA-A **(A)** and RUNX2 **(B)** in different cell type seeded or treated bone grafts.
